# Household

**Published:** 1890-05

**Authors:** 


### HOUSEHOLD.

To Disinfect a Room.—The best means to disinfect a room which has been occupied by a person suffering from any infectious disease, is to burn sulphur in the room. To do this, take a dish pan, and place a flat plate in the bottom of it, and on this plate set a kettle containing the proper amount of sulphur mixture—equal quantities of sulphur and charcoal. Fill the pan with water so that it will come half way up on the kettle. Then turn alcohol or benzine on the mixture, ignite, and get out of the room as speedily as possible. Alcohol is much the best to use, and two or three ounces will be sufficient for several pounds of sulphur. Let the room remain closed for twenty-four hours. The room should be left open for another twenty-four hours, and then thoroughly cleansed, the furniture washed with disinfectant solution, the walls newly kalsomined or papered, and the wood work covered with fresh paint.

The room should be prepared previously by having every crack about doors and windows tightly pasted or stopped up. The object of using water is that the heat of the kettle will cause evaporation and send moisture out into the room; for, the spores being very tenacious of life, dry sulphur fumes are not sufficient to kill them all. In the dry state, the product is simply oxide of sulphur, but when water is added we have sulphurous acid, which is powerful enough to kill all the spores as well as the germs.

Savory Pancake.—One large, heaped up tablespoonful of flour, a little salt and pepper to taste; mix this with half a pint of milk; add the yolks of three eggs, well beaten, also two tablespoonfuls of finely chopped parsley, and two spring onions, also finely chopped; then stir in the whites of three eggs, which must be beaten to a stiff froth, and to do this, place them on a plate, add a pinch of salt, and beat with a knife. Now the pancake is ready to fry; place some butter in the pan, and let it boil, then pour in the mixture, and let it brown; more butter must be added if the pancake seems likely to stick. When it is nicely brown (and to ascertain this, a corner must be turned up with a knife to see), hold the pan in the oven for two or three minutes, and then turn it out into a dish; the dish must not be covered when taken into the dining room.

A Useful Cement.—The following mixture has been used with the greatest possible success for the cementing of iron railing tops, iron gratings to stoves, etc.; in fact, with such effect as to resist the blows of a sledge-hammer. This mixture is composed of equal parts of sulphur and white lead, with about one-sixth proportion of borax, the three being thoroughly incorporated together, so as to form one homogeneous mass. When the application is to be made of this composition, it is wet with strong sulphuric acid, and a thin layer of it is placed between the two pieces of iron, these being at once pressed together. In five days it will be perfectly dry, all traces of the cement having vanished, and the work having every appearance of welding.

